# Bioavailability of cyanide after consumption of a single meal of foods containing high levels of cyanogenic glycosides: a crossover study in humans

**DOI:** 10.1007/s00204-015-1479-8

**Published:** 2015-02-24

**Authors:** Klaus Abraham, Thorsten Buhrke, Alfonso Lampen

**Affiliations:** Department of Food Safety, Federal Institute for Risk Assessment (BfR), Max-Dohrn-Str. 8-10, 10589 Berlin, Germany

**Keywords:** Cyanide, Cyanogenic glycosides, Bioavailability, Cassava, Linseed, Bitter apricot kernels, Persipan

## Abstract

The acute toxicity of cyanide is determined by its peak levels reached in the body. Compared to the ingestion of free cyanide, lower peak levels may be expected after consumption of foods containing cyanogenic glycosides with the same equivalent dose of cyanide. This is due to possible delayed and/or incomplete release of cyanide from the cyanogenic glycosides depending on many factors. Data on bioavailability of cyanide after consumption of foods containing high levels of cyanogenic glycosides as presented herein were necessary to allow a meaningful risk assessment for these foods. A crossover study was carried out in 12 healthy adults who consumed persipan paste (equivalent total cyanide: 68 mg/kg), linseed (220 mg/kg), bitter apricot kernels (about 3250 mg/kg), and fresh cassava roots (76–150 mg/kg), with each “meal” containing equivalents of 6.8 mg cyanide. Cyanide levels were determined in whole blood using a GC–MS method with K^13^C^15^N as internal standard. Mean levels of cyanide at the different time points were highest after consumption of cassava (15.4 µM, after 37.5 min) and bitter apricot kernels (14.3 µM, after 20 min), followed by linseed (5.7 µM, after 40 min) and 100 g persipan (1.3 µM, after 105 min). The double dose of 13.6 mg cyanide eaten with 200 g persipan paste resulted in a mean peak level of 2.9 µM (after 150 min). An acute reference dose of 0.075 mg/kg body weight was derived being valid for a single application/meal of cyanides or hydrocyanic acid as well as of unprocessed foods with cyanogenic glycosides also containing the accompanying intact β-glucosidase. For some of these foods, this approach may be overly conservative due to delayed release of cyanide, as demonstrated for linseed. In case of missing or inactivated β-glucosidase, the hazard potential is much lower.

**Electronic supplementary material:**

The online version of this article (doi:10.1007/s00204-015-1479-8) contains supplementary material, which is available to authorized users.

## Introduction

Cyanogenic glycosides are secondary plant metabolites which are present in more than 2600 species, including up to 26 economically important crops. Their ability to liberate toxic levels of hydrocyanic acid (HCN, hydrogen cyanide) principally offers an immediate chemical defence response to herbivores and pathogens causing damage to the plant tissue—if their content is high enough. Cyanogenic glycosides have gained additional functionalities as transporters of nitrogen, and operation of an endogenous turnover pathway may enable plants to withdraw the nitrogen and glucose deposited in cyanogenic glycosides for use in primary metabolism (Møller 2010; JECFA 2012). The general structure of cyanogenic glycosides consists of a reactive α-hydroxynitrile component, stabilized through conjugation with either d-glucose or gentiobiose. Cyanogenic glycosides are stored in cell vacuoles of the plant tissue, separating them from their hydrolysing enzymes, specific β-1,6-glycosidases, and hydroxynitrile lyases. In case of destruction of the plant tissue—for example, by herbivores—the β-glucosidases come in contact with cyanogenic glycosides, resulting in the enzymatic cleavage of the carbohydrate moiety. The free α-hydroxynitrile can then either be enzymatically cleaved or spontaneously dissociate to a ketone or aldehyde and HCN (JECFA 2012).

**Fig. 1:**
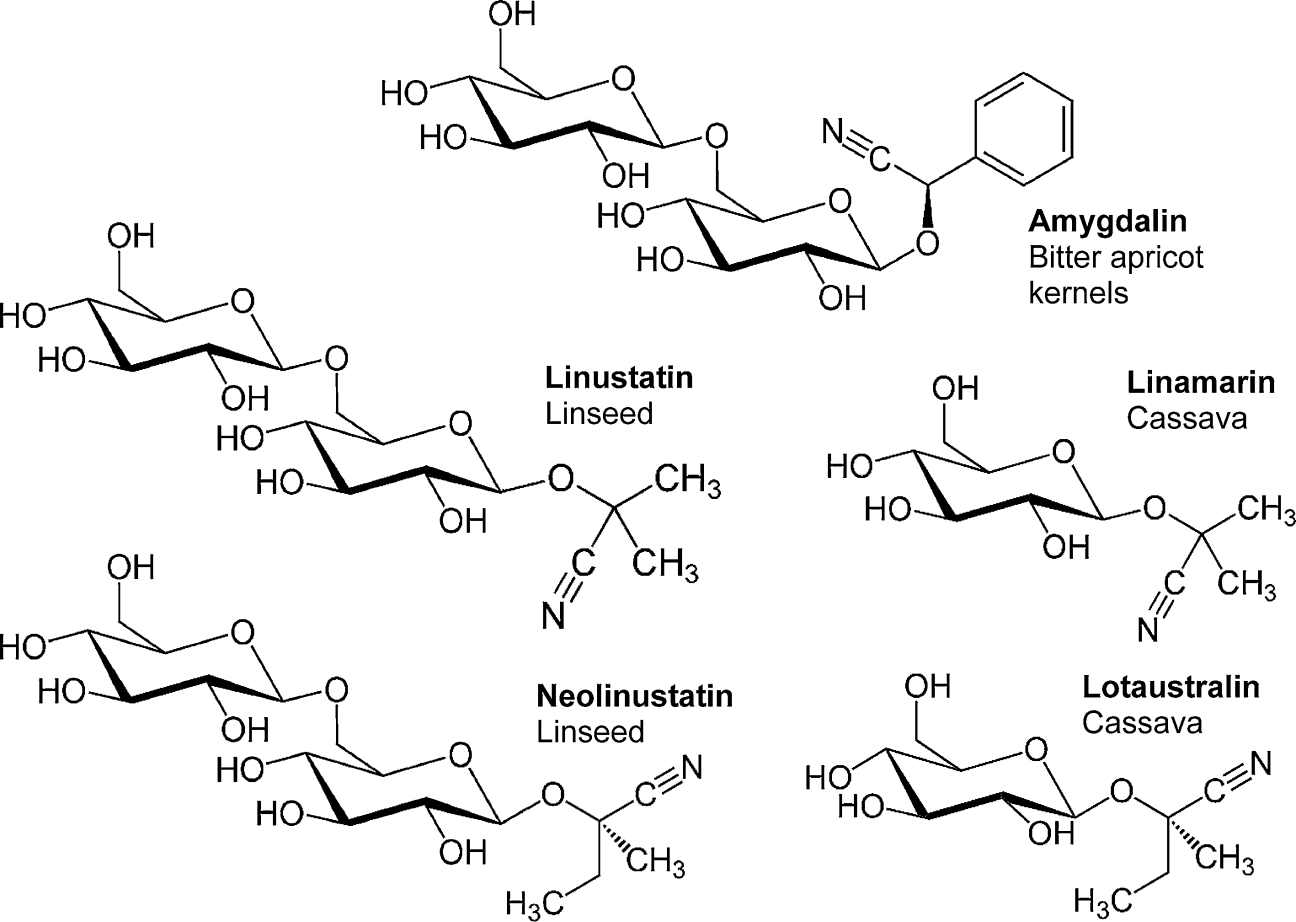
Chemical structures of the main cyanogenic glycosides in the foods investigated

The aldehyde or ketone release depends on the specific cyanogenic glycoside. Regarding the glycosides relevant to the foods used in the study presented herein (Fig. 1), benzaldehyde is released in case of amygdalin, the cyanogenic glycoside of bitter almonds and apricot kernels as well as foods produced from these kernels (for example, persipan). Benzaldehyde is responsible for the bitter taste of these foods and therefore provides a warning signal to the consumer. This is missing in case of the cyanogenic diglycosides in linseed, linustatin and neolinustatin, releasing acetone and butanone, respectively. The corresponding monoglycosides linamarin and lotaustralin are present in immature seed, but diminish to trace levels in mature seed (Barthet and Bacala 2010). However, linamarin (about 97 %) and lotaustralin (about 3 %) are the cyanogenic glycosides in cassava roots (Jørgensen et al. 2011). Following complete metabolism/hydrolysis, 1 g of linamarin and amygdalin could theoretically release 109 and 59 mg HCN, respectively. The term “total cyanide” is often used to describe the cyanide content of a food as a sum of bound (cyanogenic glycosides, cyanohydrins) and “free” cyanide. It is measured as the maximum content of cyanide releasable by hydrolysis (see “Materials and methods” section).

The (per)acute oral toxicity of cyanide ions and HCN in mammals is well known. The small molecule is readily absorbed and distributed via systemic circulation. Above a certain tissue level, cyanide inhibits the cytochrome c oxidase (complex IV)—the terminal enzyme of the mitochondrial electron transport chain—by competitively binding to the oxygen-reducing cofactor of the protein. This crucial effect causes decreased utilization of oxygen and increased anaerobic metabolism, leading to excess of lactic acid and metabolic acidosis, and finally to cell death through energy deprivation. Due to its high dependence on oxidative metabolism, the central nervous system is particularly vulnerable to cyanide intoxication. With high oral doses, symptoms occur within a few minutes and may include nausea, vomiting, giddiness, headache, palpitations, hyperpnoea then dyspnoea, bradycardia, unconsciousness, and violent convulsions, followed by death (JECFA 2012; WHO 2004). In terms of the minimal lethal dose of cyanide in humans, the number of about 0.5 mg/kg body weight is commonly cited. It dates back to a paper by Gettler and Baine (1938) who applied a formula for back-calculation of the dose—derived from experimental data in dogs—to tissue levels found in humans after lethal intoxications. Often, a range of 0.5–3.5 mg/kg body weight is cited in the literature for the acute lethal dose of cyanide in humans which first was published by Halstrøm and Møller (1945). However, in some cases, much higher doses have been survived (e.g. 2.5 g potassium cyanide equivalent to 1000 mg cyanide: Tassan et al. 1990).

Regarding possible chronic effects of cyanide, it is indeterminate whether the toxicity of cyanide is solely determined by the acute mechanisms described above, or whether a high chronic exposure to doses not causing acute toxicity may nevertheless result in health effects. In this context, the consumption of cassava roots as staple food in tropical regions with poor soil, especially in Africa, was found to be associated with chronic neurological disorders. This issue is under scientific debate since many decades, and different mechanisms are discussed besides a chronic effect of cyanide (JECFA 2012; Adamolekun 2011). The study presented herein solely focuses on acute exposure from a single meal.

Concerning cyanide detoxification in the body, approximately 80 % of absorbed cyanide is metabolized by the mitochondrial liver enzyme thiosulfate sulfurtransferase (TST, rhodanese) and by other sulphur transferases. The enzymes catalyse the transfer of sulphur from a donor to cyanide to form less toxic thiocyanate, which is readily excreted in urine. Sulphur-containing donor molecules are the rate-limiting factor in the detoxification of cyanide revealing zero-order kinetics at higher doses (JECFA 2012; WHO 2004). Moreover, the *K*_m_ value for the human rhodanese protein has been determined to be in a range of approximately 3 mM, indicating slow turnover of cyanide even in the presence of sufficient amounts of sulphur-donating molecules (Billaut-Laden et al. 2006). Several polymorphisms in the rhodanese-encoding TST gene have been identified in human populations, but only a minimal influence on cyanide detoxification was detected, some of them resulting either in diminished rhodanese activity or in a decreased level of TST gene expression (Billaut-Laden et al. 2006). Pharmacological data of sodium nitroprusside, introduced in the 1970s as intravenous drug to treat hypertensive crises, revealed the rate of spontaneous detoxification of cyanide in humans to be about 1 μg/kg body weight per min only (about 3.6 mg cyanide per h in a person with a body weight of 60 kg), more than tenfold less than estimated before (Schulz et al. 1982). Oral absorption rates of cyanide above this metabolism rate will lead to accumulation with increasing cyanide blood and tissue levels. Beginning above a certain level, this will cause increasing symptoms of acute toxicity. Therefore, the rate of absorption plays an important role in cyanide toxicity, and even an acute lethal dose is tolerated without symptoms if it is split into even parts ingested, for example, hourly over the day.

With respect to risk assessment of foods containing cyanogenic glycosides, the question arises whether it is justified to treat the glycosidic bound cyanide as it would be bioavailable in the body like free cyanide (hydrogen cyanide or cyanide ion). For the same equivalent dose of cyanide, absorption rates and resulting peak levels of cyanide in body tissues may be lower in case cyanogenic glycosides due to delayed and/or incomplete release of cyanide which requires mechanical destruction of the plant’s tissue structure and enzymatic degradation by the specific β-glucosidase which, however, may be inactivated or destructed by the acidic pH of the stomach. Mammalian tissues themselves do not contain significant amounts of specific β-1,6-glucosidase, but the bacterial flora of the gastrointestinal tract partially does (Carter et al. 1980; Newton et al. 1981). Due to the limited metabolic capacity of the rhodanese, any delay in absorption would allow time to detoxify the cyanide which would therefore also contribute to lower peak levels. These mechanisms influencing the peak levels of cyanide in body tissues can be expected to be different for different foods, depending on the degree of plant tissue destruction, the type of the cyanogenic glycoside, the effectiveness of the accompanying plant β-glucosidase, the pH value of the stomach, the bacterial flora of the gut, and/or possible influences related to the plant matrix.

With regard to the complex issue described, it is not surprising that scientific bodies have derived differing acute reference doses (ARfD) for cyanide from consumption of foods with cyanogenic glycosides, depending on the type of data (animal or human, cyanide or cyanogenic glycosides) and the safety factors used for the assessment. In 2006, the British COT derived an ARfD of 0.005 mg/kg body weight for cyanide from bitter apricot kernels, whereas the JECFA derived an ARfD of 0.090 mg/kg body weight for cyanide from foods containing cyanogenic glycosides as the main source of cyanide in 2012. Accordingly, the maximum (glycosidic bound) cyanide intake from a single meal in a person with a body weight of 60 kg would be 0.3 and 5.4 mg, respectively. For example, for marzipan with a content of 50 mg/kg this would correspond to an amount of 6 and 108 g, respectively, and for linseed with a content of 200 mg/kg to an amount of 1.5 and 27 g, respectively. These differences are unsatisfactory.

The aim of the study in humans presented herein was to generate meaningful data for the derivation of an ARfD for cyanide from ingested foods containing high levels of cyanogenic glycosides, which does not require high safety factors. Single meals of foods with high content of cyanogenic glycosides were consumed, and serial measurements of cyanide in whole blood were taken in order to identify the (absolute) peak levels serving as a surrogate marker for the peak level of cyanide in tissues triggering the acute effect of cyanide; they can be compared to clinically relevant levels known from intoxications. The investigation was performed as five-way crossover study with 12 healthy non-smoking participants, also allowing a comparison of the different foods ingested regarding their hazard potential.

Regarding the selection of study foods, they had to be relevant for risk assessment. The initial investigations were done with *persipan paste*, a sweet similar to marzipan which is common in German-speaking countries. It is in part produced from bitter apricot kernels and has a maximum cyanide level of 50 mg/kg in the European Union (EU 2008), which may be too high if the glycosidic bound cyanide is bioavailable like isolated cyanide. *Bitter apricot kernels* have—similar to bitter almonds—high contents of amygdalin (equivalent total cyanide content up to 4000 mg/kg: Zöllner and Giebelmann 2007). They have become popular in a small group of persons believing that amygdalin is mainly metabolized to toxic cyanide in cancer cells and therefore has a therapeutic and prophylactic effect on cancer (Culliton and Waterfall 1979; Milazzo et al. 2011). *Linseed* may contain cyanide equivalents of more than 200 mg/kg (CVUA Sigmaringen 2009). It is popular not only as a traditional herbal medication to treat or prevent constipation (single dose 10–15 g: EMA 2006), but also as a nutrient with high levels of α-linolenic acid (Cunnane et al. 1995). Furthermore, *fresh cassava roots* were of interest as an important staple food, especially in tropical parts of the world. They vary widely in their content of bound cyanide, although most varieties contain 15–400 mg/kg (WHO 2004). Consumption of large amounts may cause acute intoxication if the processing to reduce the content of cyanide is insufficient (Mlingi et al. 1992). In Europe, it is no common food, but offered, for example, in some Asia retail markets, and therefore may be bought by consumers not informed about the necessity of detoxification prior to consumption. This has caused concern in the European Union (Kolind-Hansen and Brimer 2010). In our study, unprocessed cassava was investigated.

## Materials and methods

### Study design

Oral bioavailability refers to the extent and the rate at which the active moiety of a compound enters systemic circulation. The acute toxicity of cyanide is triggered by its peak levels reached in tissues, determined by both parameters of absorption. After consumption of foods containing cyanogenic glycosides, monitoring of cyanide levels in blood can be used to identify the peak level serving as a surrogate marker for the peak level of cyanide in tissues. With this concept, different foods can be compared regarding the peak levels reached after consumption of foods containing the same equivalent dose of cyanide (crossover design to investigate relative bioavailability of cyanide). Being even more relevant, the peak level reached in blood is an absolute measure for the bioavailability of cyanide in terms of its toxicity, as it can be assessed in comparison with the range of clinically relevant levels known from intoxications in humans. According to Hail and Rumack (1986), first symptoms like tachycardia or flushing can be expected in the range of 0.5–1.0 mg/L (whole blood, ca. 20–40 µM); higher levels above 1.0 mg/L (ca. 40 µM) were classified as toxic (e.g. depressed consciousness), and levels higher than 2.5–3.0 mg/L (ca. 100–120 µM) were reported to be associated with life-threatening symptoms or death. According to the evaluation of Schulz et al. (1982), biochemically detectable disturbances occur at levels above 40 µM, clinically recognizable symptoms at levels above 200 µM, and possible fatalities at levels above 400 µM. These numbers were based on measurements of cyanide in erythrocytes and therefore roughly correspond to the numbers of Hail and Rumack (1986). Most of the cyanide present in blood is found in erythrocytes, with a ratio of at least 10:1 of erythrocytes to plasma (Hail and Rumack 1986). During dose-finding investigations in the medical head of the study (K.A.), a cyanide level of up to 20 µM in whole blood was considered to be safe.

The aim of the study was not only to investigate the bioavailability of cyanide after consumption of foods containing high amounts of cyanogenic glycosides, but especially to answer the question—with the background of risk assessment—whether high consumption of such foods can lead to critical levels of cyanide in blood (higher than 20 µM). For this purpose, worse case conditions with respect to resulting higher cyanide levels in blood had to be used, which are (1) highest level of cyanide equivalent (total cyanide) in a certain food available at retail, (2) maximal mechanical destruction of the tissue structure of the food immediately before ingestion or by thorough chewing, (3) fast consumption after overnight fasting (empty stomach), and (4) no other foods eaten during the first hours after consumption of the study meal. Furthermore, the amount of a certain food to be eaten had to be high in order to cover (possible) high consumption in the population, but not too high in case of those foods known to possibly cause intoxications (bitter apricot kernels, cassava). Investigations were started with 100 g persipan paste with an equivalent cyanide level of 68 mg/kg. Preliminary investigations of the medical head of the study revealed the same dose of 6.8 mg cyanide to be also suitable (not too high) for the investigations of the other foods (bitter apricot kernels, linseed, and cassava), allowing a direct comparison of these four applications. Additional investigations were made for persipan paste with the double amount of 200 g (cyanide dose 13.6 mg). A five-way crossover design involving 12 volunteers was chosen, in principle following the ‘guideline on the investigation of bioequivalence’ in case of medicinal products (EMA 2010).

### Volunteers

In order to assure high compliance, volunteers were recruited from the scientific staff of the Federal Institute for Risk Assessment. They had to be healthy non-smokers of European origin with normal body mass index. Along with the experimental blood samples taken on the first study day, extra samples were collected for measurement of liver enzymes, creatinine, and cellular blood parameters (blood count); all results obtained were within the normal ranges. The study group finally consisted of 12 participants (five non-pregnant females and seven males) with a mean age of 46 years (median 50, range 30–56), a mean height of 1.79 m (median 1.79, range 1.66–1.96), a mean body mass of 74.2 kg (median 71.5, range 60–99), and a mean body mass index of 23.0 kg/m^2^ (median 22.9, range 20.0–26.3). Mean body weight was 64.6 and 81.0 kg in the female and male participants, respectively. All participants gave informed consent in writing. The study protocol was approved by the ethics committee of the Charité—Universitätsmedizin Berlin (No. EA4/060/11 for persipan paste and No. EA4/060/12 for the other foods).

### Foods containing high levels of cyanogenic glycosides

Quantitative determination of total cyanide (sum of bound and free cyanide) in the foods examined in this study was conducted at the “Institut für Produktqualität” (ifp, Berlin, Germany) according to the official method of the Association of German Agricultural Analytic and Research Institutes for cyanide determination in feed and food (VDLUFA 1976). Briefly, 3–15 g of the respective food was homogenized using a mixer and suspended in 200 mL in 70 mM Na/K phosphate buffer with pH 5.9. After addition of 1 g freshly homogenized sweet almonds, the flask was immediately closed with a ground-in stopper, and the suspension was incubated overnight at room temperature. Subsequently, about 60 mL of the solution was distilled into 40 mL of a 1 M NaOH solution by taking advantage of water steam distillation equipment. Then, six drops of a phenol red solution, 1 mL of an aqueous 10 % potassium iodide solution, and 1 mL of an aqueous 10 % ammonia solution was added to the distillate. The solution was titrated with 10 mM silver nitrate against a black background until a first weak clouding was observed. Consumption of 1 mL of this solution was equal to the presence of 0.54 mg HCN in the distillate. Each determination was done in duplicate.

Persipan paste (batch of 12.5 kg, # 1174-07) containing 47 % bitter apricot kernels, 36 % sugar, and 17 % water (according to the product specification of the manufacturer) was a kind gift of MOLL Marzipan (Berlin, Germany), a big European producer of persipan and marzipan. Investigation of total cyanide content revealed a mean of 68 mg/kg (ten samples analysed, range 59–78 mg/kg, median 70 mg/kg, coefficient of variation 10.8 %). The content of cyanide was above the maximum level of 50 mg/kg according to European regulation (EC) No. 1334/2008 (EU 2008) for “nougat, marzipan or its substitutes, or similar products”; to conform to this regulation, a further treatment of the lot would therefore be necessary in case of selling by the manufacturer. However, the batch was accepted for the study, as one aim of the study was to investigate the safety of the maximum level mentioned. The weight of 100 g persipan paste was used for the first investigation, determining the dose of cyanide of 6.8 mg which was also used for the investigation of the other foods. In order to investigate also the safety of the double amount of persipan paste with high content of cyanide, a second investigation was performed with consumption of 200 g.

Linseed (flaxseed, *Linum usitatissimum*) from dennree GmbH (Töpen, Germany; “Leinsaat”, 500 g package, # L5202202, from Romania) was purchased at retail in Berlin. The product was found to contain the highest level of total cyanide (220 mg/kg) in a set of 15 different linseed products purchased from the German market in May 2012 (total cyanide: mean 145 mg/kg, median 131 mg/kg, minimum 98 mg/kg). The distribution of the cyanide levels found in these products was comparable to that found in an investigation of the German food control authority in Sigmaringen in 2009 (mean 154 mg/g, range 80–300 mg/kg, *n* = 38, CVUA Sigmaringen 2009) and to that of a recent investigation from New Zealand (mean 127 mg/g, range 91–178 mg/kg, *n* = 5, Cressey et al. 2013). To assure the mechanical destruction of the linseed grains, the study meal of 30.9 g (corresponding to a dose of 6.8 mg cyanide) was ground up in an analysis mill (type A10 with star-shaped cutter A13, 20,000 rpm, IKA Labortechnik, Staufen, Germany) directly before consumption; chewing the linseed would by far not be as effective with regard to destruction.

Bitter kernels of apricots (*Prunus armeniaca*) were purchased via internet from Wiessler (Wertheim, Germany; “Bio Aprikosenkerne bitter”, 500 g package, # 1967). From one package, all kernels (*n* = 1334) were weighted, revealing a mean ± SD of 0.37 ± 0.10 g (median 0.36 g, range 0.12–0.84 g). Content of total cyanide was found to be about 3250 mg/kg; for the study dose of 6.8 mg cyanide, this corresponded to a consumption of 6 medium size kernels (about 2.1 g). As a variation in the content of cyanide from kernel-to-kernel can be assumed, each study “meal” of six kernels was analysed individually. For this purpose, each of 12 kernels was split into two, using a knife to open the natural gap between the two halves of an apricot kernel. One set of the 12 halves was analysed for the content of cyanide. The result had to be in a range of 3150–3350 mg/kg; otherwise, the set was not used for the study.

Fresh cassava roots (manioc, *Manihot esculenta*) were purchased from the deli department of a large store in Berlin (originating country: Brasilia). They had a weight of several hundred grams and were stored at 4 °C until use for the study (up to 3 weeks after delivery to Berlin, by then showing no signs of post-harvest physiological deterioration like brownish discoloration: Kojima et al. 1983). Content of cyanide was found to roughly vary between 50 and 200 mg/kg. Slices of cassava roots are known to have a steep radial gradient in the content of cyanide, with highest content in the cortex (Kojima et al. 1983), reflecting the principle of chemical defence. Therefore, three peeled slices (thickness about 1 cm, weight about 30 g, taken at a longitudinal distance of a few cm) were cut from each root for the analysis of their content of cyanide. The two intermediate cylindrical pieces of the root were used for the study, if the content was found to be in the range of 75–150 mg/kg, corresponding to an amount to be eaten between 91 and 45 g, respectively (dose as for the other foods: 6.8 mg cyanide). The content of cyanide in the peeled piece to be eaten was roughly determined by interpolation of the content values obtained for the adjacent slices. The content of cassava effectively eaten by the study participants was between 76 and 150 mg/kg (mean 113 mg/kg), the corresponding mass of cassava was between 90 and 45 g (mean 64 g).

### Investigation and sample collection

Volunteers fasted overnight and came to their work place between 8.00 and 9.00 a.m. For repeated blood sampling, an intravenous catheter (Introcan Safety, B. Braun Melsungen AG, Germany) closable by a mandrin was placed in a vein of the antecubital fossa, and the first sample of heparinized whole blood was drawn (4.9 mL vial, 16 IU Li-Heparin per mL blood, Sarstedt S-Monovette 02.1065, Nümbrecht, Germany). Thereafter, participants received one of the five applications described above. They were instructed as follows: persipan paste had to be eaten in a timely manner, with chewing awhile in order to produce enough saliva for swallowing; 100 g were eaten in a mean time of 5 min (range 4–6 min), 200 g in a mean time of 12 min (range 9–13 min). The grinded linseed (30.9 g) was mixed with 70 mL of tap water, and the suspension was drunk/spooned in a mean time of 2 min (range 1–3 min). Pre-examinations had revealed this method of consumption as the one resulting in the highest blood levels of cyanide (compared to chewing of the unground or ground linseed). Bitter apricot kernels had to be chewed as thoroughly as possible (2 of the 12 halves at a time for 30 s), avoiding the swallowing of unchewed pieces of the kernels. Cassava also had to be chewed as much as possible in a timely manner; the mass of 45–90 g was eaten in a mean time of 7 min (range 5–10 min). After each consumption, the participants drunk 200 mL of tap water and a further 200 mL after 60 min; apart from this, no other foods or beverages were consumed until the end of the investigation. During the test, the participants remained in a sitting position at their workplace (low physical activity level).

The schedule for the collection of heparinized whole blood (4.9 mL, total number: 15 samples, but 18 in case of 200 g persipan paste) was adapted to the expected time span with peak levels for the different foods, with shorter intervals during this period. The shortest intervals of blood collection were 5 min [bitter apricot kernels, from start of the consumption (=0 min) to 30 min], 7.5 min (cassava, from start to 60 min), 10 min (linseed, from start to 60 min), and 15 min (persipan paste, from 60 to 180 min). The total period of collection was 3 h, but 4 and 5 h in case of 100 and 200 g persipan paste, respectively. Blood samples were stored at room temperature until sample preparation. The study was performed between October 2011 and August 2013. The interval between two tests in each volunteer was at least 2 weeks.

### Measurement of cyanide in whole blood

All chemicals were purchased from Sigma-Aldrich (Munich, Germany) or Merck (Darmstadt, Germany) at the highest purity available. Isotopically labelled internal standard K^13^C^15^N was purchased from Sigma-Aldrich (# 490539). GC–MS analysis was conducted by using an Agilent 7890A gas chromatograph coupled to an Agilent 5975C mass spectrometer (Agilent, Böblingen, Germany). The system was equipped with an MPS2 autosampler controlled by the Maestro software (Gerstel, Mülheim, Germany).

Sample preparation done in duplicate was started within 1 h after blood sampling. A volume of 0.1 mL of a 0.1 M NaOH solution containing 100 µM K^13^C^15^N was pipetted into a 20 mL headspace vial. Subsequently, 0.2 mL of a 0.9 % NaCl solution containing 1 M ascorbic acid was added to the vial followed by the immediate addition of 1 mL of a whole blood sample. The vial was closed with a septum without crimping and was immediately put into a −80 °C freezer for at least 15 min. Then, 0.2 mL of a 1:1 mixture of 85 % phosphoric acid and distilled water was added to the frozen sample; the vial was finally closed, crimped, and the sample was thawed at room temperature and then vortexed for 10 s. Finally, it was stored at 4 °C until GC–MS analysis carried out within 2 days. A time of storage up to 1 week was found not to influence the results of the measurement.

Cyanide determination was conducted according to the GC–MS method published by Dumas et al. (2005). Each sample was incubated for 15 min at 60 °C and 600 rpm by taking advantage of the incubator belonging to the MPS2 autosampler. Using a 2.5-mL headspace syringe at a constant temperature of 80 °C, 1.5 mL of the gas phase was injected at 50 µL/s into the injector set to 130 °C. The splitless mode was used, and the septum flow was closed during sample injection. The sample was separated on a 30-m GS-GasPro column (# 113-4332; Agilent). Helium was used as the mobile phase at a constant flow rate of 2.1 mL/min. The oven program was 40 °C for 2 min followed by a gradient of 25 °C/min up to 240 °C, and a final bake-out for 5 min at 240 °C. The duration of each run was 15 min, and HCN had a retention time of about 7 min under the given conditions. The temperature of the transfer line and of the MS ion source was 230 °C, and the quadrupole was set to 150 °C. To increase sensitivity of the instrument for low masses, a manual tuning was conducted after the standard auto-tune procedure. For this purpose, the internal calibrant PFTBA was turned off, and the emission current, the repeller and the lens parameters were optimized for the masses *m*/*z* 18 (water), *m*/*z* 32 (oxygen), and *m*/*z* 40 (argon).

In order to avoid carry-over between two injections as reported by Dumas et al. (2005), the needle of the syringe was flushed with nitrogen for 10 min after each injection. Moreover, a control measurement was taken always between the injections of two samples by injecting 1 mL of air into the system. This control sample was run using the same GC–MS program in order to demonstrate the lack of a cyanide signal in the gas chromatogram obtained from this control injection of air.

The internal standard was found to contain approximately 1 % of unlabelled cyanide. Therefore, 1 % of the peak intensity of *m*/*z* 29 (H^13^C^15^N) was subtracted from the peak intensity of *m*/*z* 27 (HCN) and added to the peak intensity of *m*/*z* 29. Then, the concentration of unlabelled HCN was calculated from the mass ratio 27/29, thereby taking into account that each sample contained 10 µM of the internal standard. Finally, the values were corrected by the response factor (*R*_f_), which were found to be in a range between 0.87 and 0.93 depending on the GC column in use. The method was calibrated by measuring spiked whole blood samples containing different concentrations of unlabelled potassium cyanide in the range between 0 and 20 µM. The method was linear within this range (and up to 50 µM), and the limit of detection (LOD) was 0.3 µM, the limit of quantitation (LOQ) was 1.2 µM, and the inter-run coefficient of variation (*V*_0_) was 2.5 %.

### Additional investigations in the medical head of the study

Further investigations were made by the medical head of the study (K.A., body weight about 80 kg, height 184 cm; referred to as test person No. 5 in the following). In order to investigate the dose dependency of the cyanide peak levels in blood, 7.5, 15, 30.9, 60, and 100 g linseed were eaten. The last two applications took a longer duration of 6 and 13 min, respectively, to completely eat these “meals”. Likewise, with the aim to investigate the safety in case of a very high consumption, 400 g persipan paste was eaten, which took little more than 1 h. Furthermore, 100 g persipan paste was eaten together with “sweet” almonds (10 and 30 g, respectively) from California containing specific β-glucosidase, but no cyanogenic glycosides. Sampling period usually was 3–5 h, but 8 h in case of consumption of 400 g persipan paste.

To compare the results obtained for foods with high content of cyanogenic glycosides, bioavailability of cyanide was also investigated after application of the free compound (6 or 17.0 mg potassium cyanide, corresponding to 2.4 or 6.8 mg cyanide, respectively, dissolved in 40 mL tap water). Furthermore, the isolated cyanogenic glycosides amygdalin and linamarin were administered in a capsule not resistant to gastric acid. Amygdalin (CAS No. 29883-15-6) was purchased from the Flora Apotheke (Hannover, Germany) in capsules containing 500 mg (pharmaceutical quality, # 05120). Linamarin (CAS No. 554-35-8) was purchased from Carbosyth (Compton, UK; purity >98 %, # ML045511101). Equivalent doses of 6.8 and 22.0 mg cyanide were administered as amygdalin (120 and 387 mg, respectively) and linamarin (64.7 and 209 mg, respectively). Additionally, 120 mg amygdalin was applied together with 10 g sweet almonds. Sampling period was 3–5 h.

### Genotyping of the TST gene

Genotyping of the TST gene of the 12 volunteers was conducted by Eurofins Genomics (Ebersberg, Germany). Genomic DNA was isolated from whole blood samples using the NucleoSpin 96 Food kit (Macherey-Nagel, Düren, Germany) according to the manufacturers’ instructions. The coding region of the TST gene as well as approximately 1 kb of the upstream region was amplified using the primers and the PCR conditions listed in Supplementary Tables 1 and 2. Sequencing of the PCR products was carried out by internal protocols of Eurofins Genomics.

### Evaluation and statistics

Standard statistical evaluation procedures were performed using Microsoft^®^ Office Excel 2003 and SPSS version 12.0. Details of the operations used are described under “Results” section.

## Results

### Concentration–time curves of cyanide in blood

**Fig. 2:**
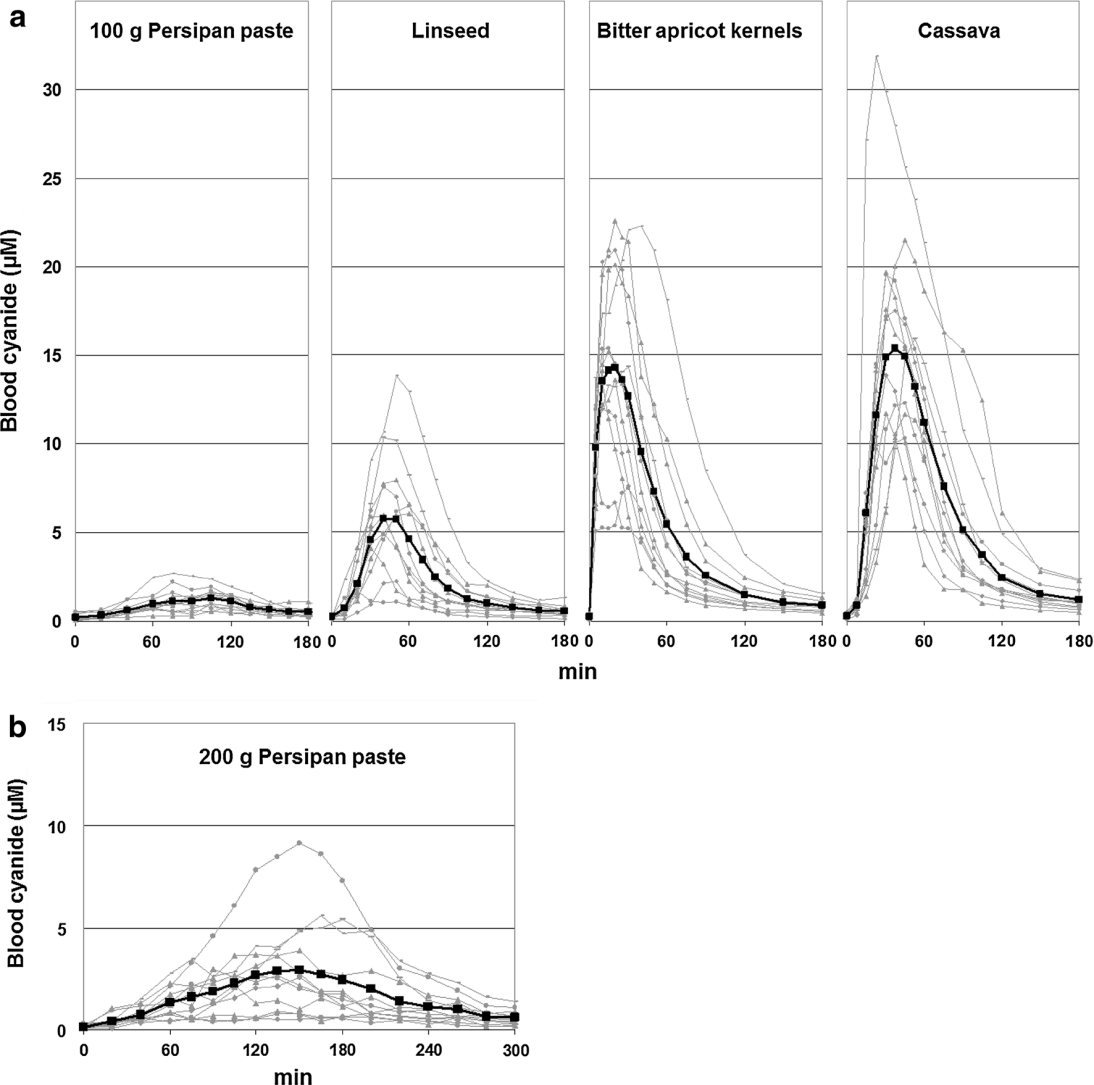
**a** Concentration–time curves of cyanide in whole blood after consumption of 100 g persipan paste, 30.9 g linseed, about 2.1 g bitter apricot kernels, and 76–150 g unprocessed cassava (equivalent dose of cyanide: 6.8 mg each). Individual curves of the 12 participants are displayed in *grey*; the curve of the means is displayed in *black* (*bold*). **b** Concentration–time curves of cyanide in whole blood after consumption 200 g persipan paste (equivalent dose of cyanide: 13.6 mg). Individual curves of the 12 participants are displayed in *grey*; the curve of the means is displayed in *black* (*bold*)

The individual concentration–time curves observed after ingestion of the four foods (persipan paste, bitter apricot kernels, linseed, and cassava) are displayed in Fig. 2a (graph of the means in bold). Mean levels of cyanide at the different time points were highest after consumption of cassava (*C*_max_ 15.4 µM, after 37.5 min) and bitter apricot kernels (14.3 µM, after 20 min), followed by linseed (5.7 µM, after 40 min) and 100 g persipan (1.3 µM, after 105 min). All these “meals” contained the same equivalent dose of 6.8 mg cyanide. The double dose eaten with 200 g persipan resulted in a peak level of 2.9 µM (mean after 150 min). Concentration–time curves are shown in Fig. 2b.

Statistical evaluation of individual *C*_max_ values is shown in Table 1 (left part). Possibly, critical cyanide levels above 20 µM were reached in four individuals after consumption of bitter apricot kernels (maximum: 22.5 µM) and in two of these individuals after consumption of cassava (21.5 and 31.9 µM, respectively). No clinical symptoms of cyanide intoxication were observed.

**Table 1:** Statistical evaluation of individual *C*_max_ values for the different applications, as well as mean values (±SD) differentiated according to sex: f = females (*n* = 5), m = males (*n* = 7)

	Mean ± SD (µM)	Min. (µM)	Max. (µM)	Median (µM)`	Sex	Mean ± SD (µM)	Significance^a^ *p* value
Persipan 100 g	1.44 ± 0.60	0.61	2.72	1.48	F	1.49 ± 0.80	n.s.
					M	1.41 ± 0.49	
Persipan 200 g	3.40 ± 2.38	0.78	9.12	2.65	F	3.44 ± 2.20	n.s.
					M	3.37 ± 2.68	
Linseed	6.40 ± 3.34	1.69	13.85	5.97	F	9.15 ± 3.04	<0.05
					M	4.44 ± 1.85	
Bitter apricot kernels	15.46 ± 5.12	7.48	22.59	14.75	F	20.06 ± 3.35	<0.01
					M	12.17 ± 3.19	
Cassava	16.95 ± 5.96	10.31	31.87	16.72	F	21.30 ± 6.28	<0.05
					M	13.84 ± 3.44	

*n.s.* not significant

^a^ *t* test for independent samples, assuming unequal variances

### Comparison of the different applications

Comparison of *C*_max_ and *t*_max_ values for two applications at a time (paired *t* test) revealed significant differences in case of 100 and 200 g persipan paste and in case of the four different foods with an equivalent dose of 6.8 mg cyanide (*p* < 0.01), but not for the comparison of bitter apricot kernels and cassava. Correlation analysis of *C*_max_ values (Pearson) revealed high correlation between 100 and 200 g persipan paste (*R* = 0.81, *p* < 0.01), which also indicates a high total reproducibility of the results obtained in individuals. Regarding the four different foods with the same equivalent dose of 6.8 mg cyanide, highest correlations of *C*_max_ values were observed in case of linseed and cassava (*R* = 0.81, *p* < 0.01) and in case of bitter apricot kernels and cassava (*R* = 0.76, *p* < 0.01), followed a slightly lower correlation in case of linseed and bitter apricot kernels (*R* = 0.69, *p* < 0.05). No significant correlations of *C*_max_ values were observed in case of 100 g persipan and each of the other three foods; however, correlations were higher for persipan and linseed (*R* = 0.51) and for persipan and cassava (*R* = 0.56), compared to persipan and bitter apricot kernels (*R* = 0.10). The corresponding correlation analysis of *t*_max_ values revealed no significant results. Likewise, no significant correlations were observed between *t*_max_ and corresponding *C*_max_ values of the same application.

### Factors possibly influencing individual *C*_max_ values

Correlation analysis of *C*_max_ values (Pearson) revealed negative correlation with body weight in case of bitter apricot kernels (*R* = −0.75, *p* < 0.01), cassava (*R* = −0.58, *p* < 0.05), and linseed (*R* = −0.57, n.s.), whereas no relevant correlation was observed in case of 100 and 200 g persipan paste (*R* = −0.13 and 0.14, respectively). Significant correlation may also be due to an influence of the sex of the participants, as mean body weight in females (64.6 kg, range 60–71, *n* = 5) was lower than in males (81.0 kg, range 60–99, *n* = 7). Multivariate regression analysis (linear regression with SPSS) with *C*_max_ as independent and with body weight and sex as dependent parameters revealed a stronger influence of sex compared to body weight in all three cases with relevant correlation. In Table 1 (right part), statistical evaluation of *C*_max_ (mean ± SD) for the different applications is given separately for females and males, showing large differences in case of linseed, bitter apricot kernels, and cassava. These differences were tested to be significant (two-sided *t* test). The age of the participants was not found to significantly correlate with *C*_max_ for any of the different applications.

### Additional investigations of the medical head of the study

**Fig. 3:**
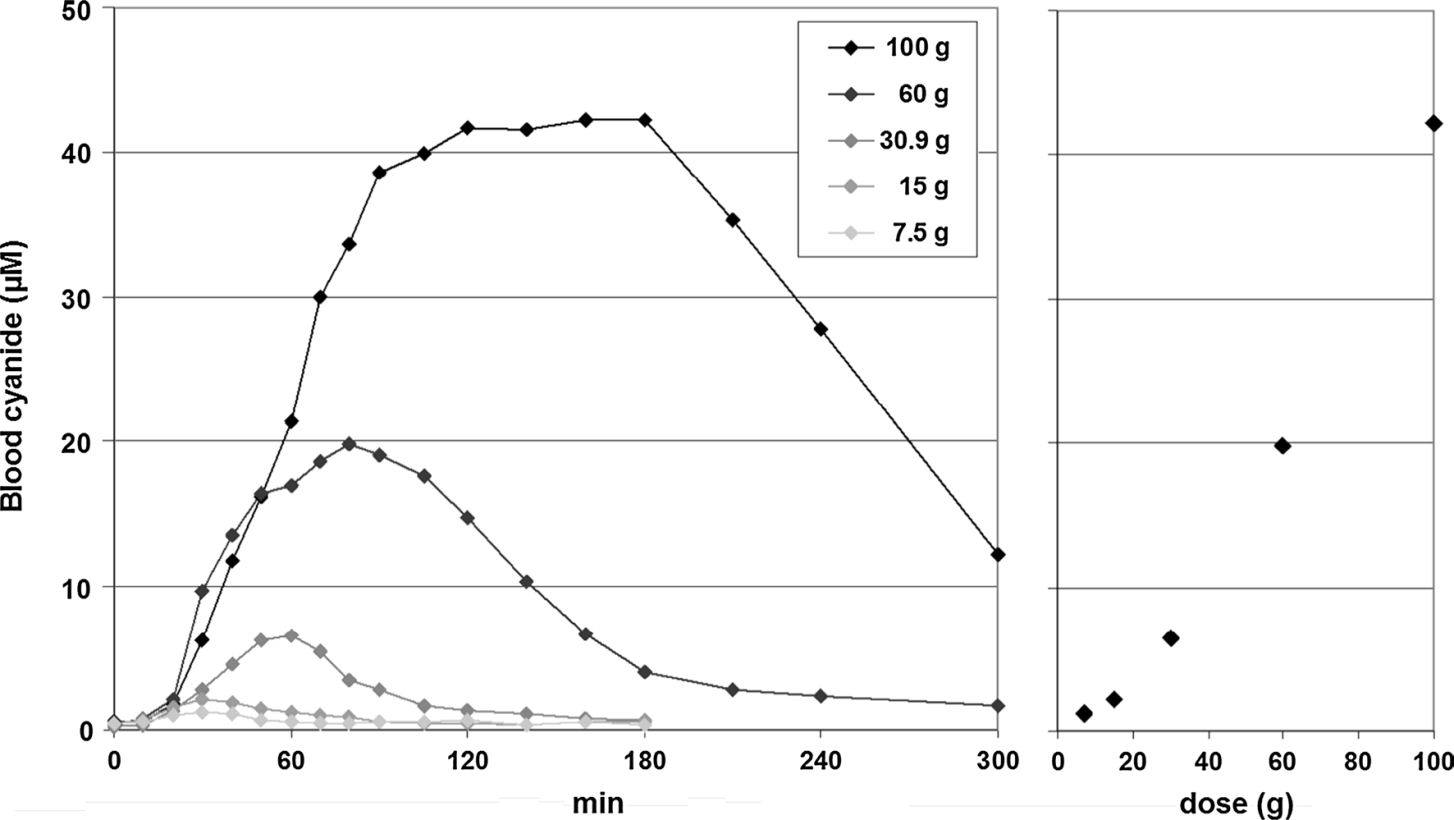
Dose dependency of the concentration–time curves of cyanide in whole blood after ingestion of different amounts of linseed (7.5–100 g with a cyanide content of 220 mg/kg) by test person No. 5 (*left side*). On the *right side*, the peak levels are displayed versus the dose of linseed

In order to investigate the dose dependency of the cyanide peak levels in blood, 7.5, 15, 30.9, 60, and 100 g linseed were eaten by test person No. 5, corresponding to equivalent doses of 1.7, 3.3, 6.8, 13.2, and 22 mg cyanide. The values for *C*_max_ measured in blood were 1.2, 2.2, 6.5, 19.8, and 42.3 µM, respectively, with corresponding *t*_max_ values of 30, 30, 60, 80, and 160 min, respectively (Table 2). No clinical symptoms of cyanide intoxication were observed. The concentration–time curves of the different doses are shown in Fig. 3 (left), and the dose dependency of the *C*_max_ values in Fig. 3 (right), revealing an overproportional increase.

**Table 2:** Results for the peaks of cyanide in whole blood (*C*_max_, *t*_max_) after application of different foods, potassium cyanide, and isolated cyanogenic glycosides in test person No. 5

Food	Amount ingested (g)	+ sweet almonds (g)	Equiv. dose of cyanide (mg)	*C*_max_ (µM)	*t*_max_ (min)
Cassava	62.0^a,c^		6.8	19.5	30
Bitter apricot kernels	2.1^a,c^		6.8	15.4	15
Linseed	7.5		1.7	1.2	30
	15.0		3.3	2.2	30
	30.9^a^		6.8	6.5	60
	60.0		13.2	19.8	80
	100.0		22.0	42.3	160
Persipan paste	100.0^a,c^		6.8	2.3	75
	100.0^c^	10.0	6.8	4.5	30
	100.0	30.0	6.8	3.4	50
	200.0^a,c^		13.6	9.1	150
	400.0^c^		27.2	17.1	270
KCN	0.006		2.4	6.0	5
	0.017^c^		6.8	20.1	10
Amygdalin	0.120^c^		6.8	3.4	60
	0.120^c^	10.0	6.8	10.0	30
	0.387		22.0	29.2	70
Linamarin	0.065^c^		6.8	0.4	–^b^
	0.209		22.0	0.9	–^b^

^a^Results obtained during the investigations within the study protocol for comparison

^b^No definite peak identifiable

^c^For concentration–time curves see Fig. 4 or 5

**Fig. 4:**
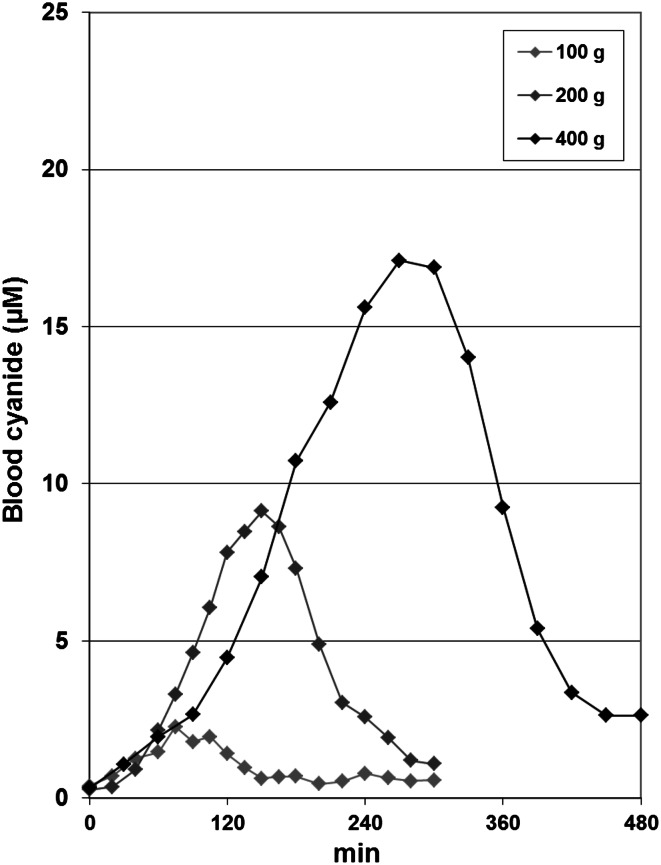
Dose dependency of the concentration–time curves of cyanide in whole blood after ingestion of different amounts of persipan paste (100, 200, and 400 g with a cyanide content of 68 mg/kg) by test person No. 5

**Fig. 5:**
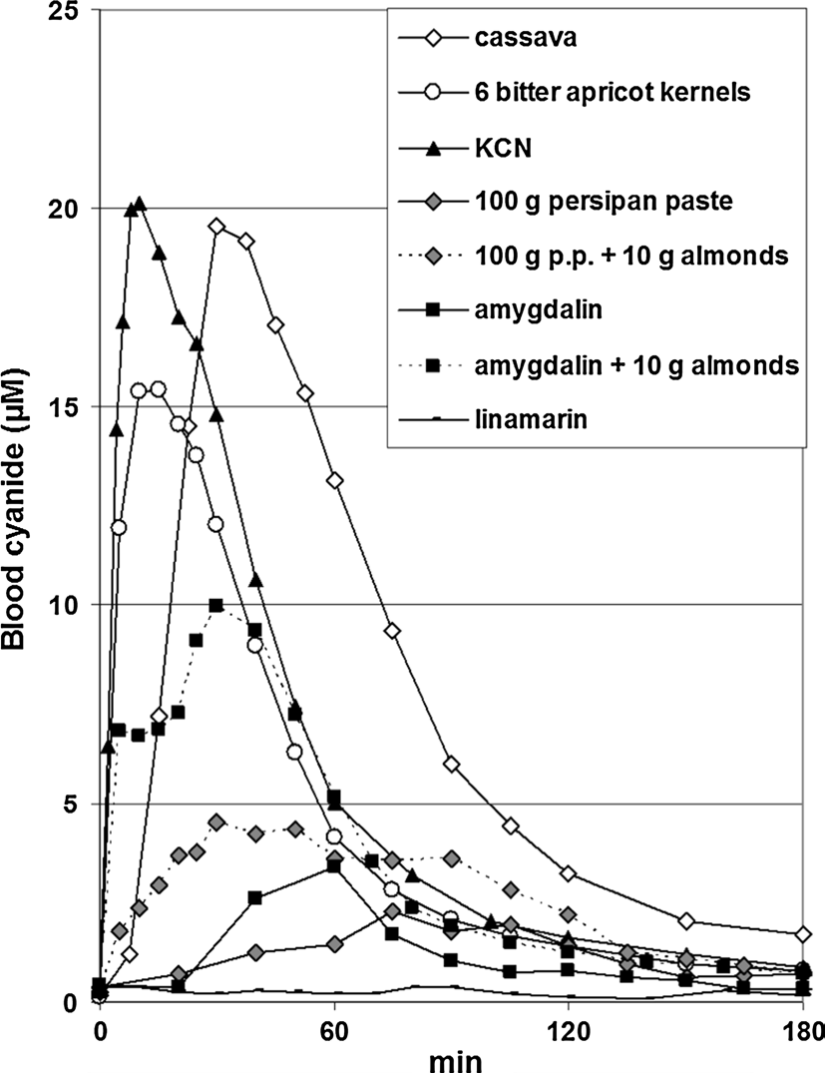
Concentration–time curves of cyanide in whole blood after ingestion of different foods, amygdalin, and linamarin by test person No. 5 (equivalent dose of cyanide: 6.8 mg each, *n* = 1 each)

Following consumption of 400 g persipan paste, a maximum cyanide level in blood of 17.1 µM was observed after 270 min; the late peak is partly due to the fact that consumption took more than 1 h time. After 480 min, the level was reduced to 2.6 µM. *C*_max_ values observed for the study doses of 100 and 200 g persipan paste were 2.3 µM (*t*_max_ 75 min) and 9.1 µM (*t*_max_ 150 min), respectively. Concentration–time curves are displayed in Fig. 4. The simultaneous consumption of 10 or 30 g sweet almonds together with 100 g persipan paste resulted in higher peak levels of 4.5 µM (*t*_max_ 30 min) and 3.4 µM (*t*_max_ 50 min), respectively. After ingestion of 120 mg amygdalin (6.8 mg cyanide), a peak level of cyanide of 3.4 µM was reached, which could be increased to 10.0 µM in case of the simultaneous ingestion of 10 g sweet almonds. In contrast, after ingestion of 65 and 209 mg linamarin (6.8 and 22 mg cyanide, respectively), no definite increase in cyanide levels in blood was observed. The application of a solution of 17 mg potassium cyanide (6.8 mg cyanide) resulted in a peak level of 20.1 µM after 10 min. These and further results are complied in Table 2, and in part displayed in Fig. 5.

For the overall interpretation of these and other results, it should be noted that test person No. 5—in comparison with the other participants of the study—was found to have relatively high peak values following the applications of persipan paste (highest value for 200 g), but not in case of the three other study foods (“ranging” place four or five).

### Alterations in the TST gene sequence of the twelve volunteers

TST genotyping did not give any indication for a mutation in the TST gene region of any of the volunteers that had to be considered in the evaluation of the study results (for details, see Supplementary Material).

## Discussion

### Concept and methods

The aim of the study was to generate meaningful data for risk assessment of foods containing high levels of cyanogenic glycosides. The (per)acute toxicity of cyanide is triggered by its tissue levels, and serial measurements of cyanide in blood in order to identify the peak reached after consumption of such foods are a useful concept, as these (absolute) peak levels can be compared to clinically relevant levels observed in intoxications of humans. In this special type of toxicity, the total amount of cyanide absorbed is not relevant in terms of toxicity, and therefore, quantitative measurements of urinary thiocyanate excretion as marker of exposure were not included. The study concept further allowed the comparison of the different foods ingested with the same equivalent dose of cyanide, allowing an evaluation of relative bioavailability.

Comparable studies are missing besides those of Oluwole et al. (2002) and Schulz et al. (1983). The latter authors investigated a group of 10 participants who consumed a meal of 30 g linseed; furthermore, a single person even ingested 100 g linseed, 10 or 50 bitter almonds, or solutions of 3, 6, or 12 mg potassium cyanide. While the dose-dependent levels of cyanide in erythrocytes were plausible for potassium cyanide with peak levels after 10–20 min, corresponding to those in whole blood observed in test person No. 5 (Table 2; Fig. 5), no relevant increase in blood cyanide was observed after consumption of linseed or 10 bitter almonds. The latter obviously misled the single person to eat 50 bitter almonds which resulted in a peak level of about 160 µM, a potentially lethal concentration (Schulz et al. 1983). Possibly, the authors have faced analytical problems. Oluwole et al. (2002) investigated a group of 12 subjects after consumption of a single meal of 150 g processed cassava (gari), but observed only a doubling of cyanide baseline levels in plasma 8 h later, starting from a mean baseline level of cyanide in plasma of about 6 µM. This would correspond to even higher levels in whole blood, as most of the cyanide present in blood is found in erythrocytes, with a ratio of at least 10:1 of erythrocytes to plasma (Hail and Rumack 1986; Lundquist et al. 1985). In our group of 12 non-smokers, baseline levels of cyanide in whole blood were 0.22 ± 0.13 µM (mean ± SD, range 0.03–0.57 µM, *n* = 60 measurements), comparable to those of others (e.g. 0.13 ± 0.08 µM, using a different analytical method: Lundquist et al. 1985). The GC–MS method using K^13^C^15^N as internal standard applied in our study (Dumas et al. 2005) was found to be very reliable, providing reproducible results.

### Bitter apricot kernels and cassava roots

Highest peak levels of cyanide were observed after ingestion of bitter apricot kernels and fresh cassava. Obviously, the reaction between the cyanogenic glycoside and the accompanying β-glucosidase of the plant is very fast and effective after destruction of the tissue structure by chewing and is not significantly impaired by the lower pH of the stomach. In case of bitter apricot kernels, the reaction is so fast that cyanide evaporating from the mouth can be smelled by a person standing next to the test person, leading to partial loss of cyanide released. The on-average later occurrence of the peak level in case of cassava is in part due to the longer duration of chewing (mean difference 4 min). Nevertheless, *C*_max_ and *t*_max_, respectively, were not found to be significantly different for the two applications. Furthermore, individual peak levels for bitter apricot kernels and for cassava revealed a high correlation, indicating comparable mechanisms determining the peak levels.

The intake of a potassium cyanide solution by test person No. 5, using the same dose of 6.8 mg cyanide, resulted in a peak level of 20.1 µM (after 10 min), which is only little higher than his peak levels after ingestion of bitter apricot kernels (15.4 µM after 15 min) and cassava (19.5 µM after 30 min, see also Fig. 5). This confirms a fast and more or less complete enzymatic release of cyanide from the cyanogenic glycosides in bitter apricot kernels and cassava, started by thorough chewing of these foods. Moreover, significant negative correlations were found between individual peak levels of cyanide and body weight in case of bitter apricot kernels and cassava, respectively. This also indicates a fast absorption of the same individual dose, with individual peak levels in blood defined by the volume of distribution, primarily determined by the individual body weight. Obviously, metabolism and excretion are of minor importance for peak levels of cyanide occurring shortly after ingestion.

A fast and more or less complete release of cyanide by the accompanying linamarase after ingestion of unprocessed cassava seems to disagree with the observation in human studies showing a significant proportion of the ingested linamarin detectable unmetabolized in urine (up to 28 %, as compiled in JECFA (2012)). However, these studies were performed using processed cassava, expected to contain much less linamarase compared to the fresh and unprocessed cassava used in our study.

### Persipan paste

Lowest peak levels of cyanide in blood were observed after consumption of 100 g persipan paste, more than 10 times lower compared to bitter apricot kernels and cassava with the same dose of cyanide. This is primarily due to missing β-glucosidase: at the end of the production process, the paste is heated to temperatures slightly above 100 °C (information by MOLL Marzipan), leading to inactivation of the β-glucosidase occurring above 75 °C (Hanssen and Sturm 1967). Therefore, specific β-glucosidase activity for cleavage of cyanide can only be provided by the bacterial flora of the gastrointestinal tract (Carter et al. 1980; Newton et al. 1981). Due to the high caloric content (460 kcal per 100 g according to the product specification), persipan paste takes time to get there due to slow emptying of the stomach. Indeed, increasing the dose from 100 to 200 g resulted in a distinctly higher *t*_max_ (105 vs. 150 min, respectively; see Fig. 2a, b). After the consumption of 400 g by a single person, the peak level of cyanide was observed even after 270 min (Table 2). According to general mechanisms in pharmacology, any delay in absorption leads to lower peak values and allows the body time to detoxify the cyanide in this case of limited metabolic capacity of the rhodanese, also contributing to lower peak levels. These peak levels were not found to negatively correlate with body weight (as observed for the other foods), and the relationship of individual capacity of β-glucosidase (bacterial flora) and of rhodanese is expected to be the main factor determining individual peak levels of cyanide. Taking all together, it is not possible to reach critical peak levels of cyanide in case of marzipan or persipan with missing activity of specific β-glucosidase and an equivalent cyanide content of up to 50 mg/kg (maximum regulatory level of the European Union, EU 2008), even in case of “as much you can eat” conditions.

The importance of a functional specific β-glucosidase is underlined by the results of test person No. 5 obtained after consumption of 100 g persipan paste together with sweet almonds, containing the enzyme but no cyanogenic glycosides. The highest peak level of cyanide in blood (4.5 µM after 30 min, with 10 g almonds) was about double as high compared to that after consumption of persipan paste only (2.3 µM after 75 min). With the higher amount of 30 g almonds, the peak level did not increase further (3.4 µM after 50 min), probably due to a less optimal molar relation of glycoside and enzyme and due to the higher total mass ingested leading to a delayed transport in the gastrointestinal tract. Thus, simultaneous consumption of β-glucosidase-containing foods increases the release of free cyanide from persipan paste; however, the peak levels of cyanide in blood were much lower than those obtained after consumption of the same dose eaten as bitter apricot kernels.

### Isolated cyanogenic glycosides

Regarding cyanide kinetics, the consumption of the isolated cyanogenic glycoside, amygdalin, could be expected to be similar to that of persipan paste, except the missing caloric load allowing a faster gastrointestinal transport. Indeed, amygdalin in a capsule with the same dose of bound cyanide caused a peak level (3.4 µM) only little higher than that of persipan paste (2.3 µM, data of test person No. 5). Simultaneous consumption of 10 g sweet almonds resulted in a much higher peak level of 10.0 µM. These results correspond to those obtained in two cancer patients orally treated with a dose of 500 mg amygdalin (bound cyanide: 28.5 mg) three times a day. After addition of 30 g almonds to the breakfast consumed 1 h after the morning dose, much higher cyanide peak levels were observed in blood, in one case accompanied by symptoms of cyanide intoxication at a cyanide level of 2.01 mg/L (74.4 µM) 2 h after amygdalin administration (Moertel et al. 1981).

Surprisingly, no corresponding increase in cyanide blood levels was observed after administration of the isolated cyanogenic glycoside of cassava, linamarin, even at an equivalent dose of 22 mg cyanide. In contrast, the same dose of bound cyanide given as amygdalin caused a peak level of 29.2 µM (data of test person No. 5). These results are differing from those obtained in hamsters not showing such a high difference of blood cyanide levels after oral administration of amygdalin or linamarin (Frakes et al. 1986). Furthermore, high doses of isolated linamarin were able to kill hamsters (Frakes et al. 1986) as well as rats (Barrett et al. 1977). No reports on the fate of isolated linamarin after oral administration in humans are available. Therefore, it remains unresolved whether the observation in test person No. 5 can be generalized or not. Individual differences in the composition of the gut flora may contribute to differences in the release of cyanide from amygdalin or linamarin depending on the activity of bacterial β-glucosidases.

### Linseed

With respect to peak levels of cyanide in blood, the results obtained after ingestion of linseed were in between those obtained for bitter apricot kernels and cassava on the one hand and those obtained for persipan paste and isolated amygdalin on the other hand. Obviously, the effectiveness of the β-glucosidase of linseed is much lower compared to that of bitter apricot kernels and cassava. This is supported by in vitro results showing a maximum of cyanide level released by autohydrolysis 2–3 h after homogenization of different cultivars of linseed (Chadha et al. 1995). Similar in vitro experiments were performed with the foods of this study, confirming the much faster release of cyanide after destruction of the plant tissue in case of bitter apricot kernels and cassava compared to linseed (Schneider et al. 2014; details will be published separately). The release of cyanide by the β-glucosidase may also be negatively influenced by the low pH of the stomach; this possible influence, however, was not quantifiable in the study.

The relatively slow release of cyanide after ingestion of freshly grinded linseed results in a lower hazard potential. Indeed, no reports on cyanide poisoning after consumption of linseed were found in the literature. The ingestion of different doses of the study linseed (7.5–100 g) by test person No. 5 resulted in peak levels of cyanide overproportionally increasing with increasing doses (Fig. 3), caused by the constant metabolism rate (at higher doses) and occurring despite increasing *t*_max_ values allowing more time for detoxification. With the highest dose, the cyanide peak level of 42.3 µM reached may potentially be associated with first clinical signs of toxicity. However, this is a very high amount of linseed hard to ingest quickly (caloric content 370–380 kcal per 100 g), and in order to meet worst-case conditions, it has to be eaten on an empty stomach directly after grinding by a machine (chewing of the hard seeds is not effective enough and very time-consuming), without consumption of other foods. In Sweden, the highest daily dose was reported to be 80 g ground linseed, given as “fibre shock” in a private health spa (Rosling 1993). Usually, high doses are up to 15 g three times a day in case of traditional herbal medication to treat or prevent constipation (EMA 2006), and this dose is safe with respect to possible acute toxicity of cyanide. Only concentrations of bound cyanide much higher than those of our study linseed (220 mg/kg) would change this assessment.

### Risk assessment (ARfD for cyanide)

As described above, the range of cyanide levels in whole blood of 20–40 µM was estimated to be (possibly) associated with first clinical symptoms of intoxication. In our study, even the highest cyanide levels in blood (42.3 µM in test person No. 5 after 100 g linseed and 31.9 µM in test person No. 9 after cassava) were not accompanied be clinical symptoms. This may be due to higher variability especially with respect to dynamics, and/or a general tendency to monitor relatively low blood levels in case of cyanide intoxications, as the peak level occurring shortly after ingestion most often will be missed due to late start of monitoring. Nevertheless, the internal cyanide level of 20 µM in whole blood (and not a higher value) was used as a threshold which should not be exceeded; accordingly, this approach contains a conservative element.

In our study, the highest peak level was 31.9 µM (after consumption of cassava) in test person No. 9 who had the lowest body weight of the study group of 60 kg. The equivalent dose of 6.8 mg cyanide therefore corresponded to the highest individual dose of 0.113 mg/kg body weight. Roughly, 2/3 of this dose (0.075 mg/kg body weight) would have resulted in a cyanide peak level of 20 µM in this test person. The application of any further safety factors was not judged to be necessary, as peak levels were found to be mainly determined by the body weight defining the volume of distribution, whereas mechanisms of elimination (e.g. activity of rhodanese, with possible higher variability) are unimportant for peak levels in case of a bolus application of a relatively high dose of cyanide. Therefore, the ARfD derived is 0.075 mg/kg body weight, corresponding to an absolute dose of 4.5 mg cyanide in a person with a body weight of 60 kg. The value is also supported by the following theoretical calculations: using the approximation of Schulz et al. (1982) that cyanide is distributed in the body to the extent of about 40 % in the blood and 60 % in the tissues, the ARfD would correspond to a dose of 0.030 mg/kg body weight in blood, which has a volume of about 0.090 L/kg body weight. This results in a maximum cyanide level of 0.33 mg/L whole blood lower than the value of 0.5 mg/L whole blood (ca. 20 µM) used as a threshold for the internal dose.

The ARfD of 0.075 mg/kg body weight is near to that of JECFA (2012) of 0.090 mg/kg body weight derived recently, using a developmental study with a single oral dose of isolated linamarin in pregnant hamsters on day 8 of gestation (endpoint: increased proportion of skeletal defects in the foetuses: Frakes et al. 1985), as well as benchmark modelling and a total safety factor of 100. This approach, however, is not convincing for us, as the effect is not the typical (per)acute one of cyanide. More importantly, the effect after oral application of isolated linamarin is expected to be much lower compared to an application together with β-glucosidase (as in fresh cassava, see study results). On the other hand, the very low ARfD of 0.005 mg/kg body weight derived by COT (2006) using human data is overly conservative in our view, as the second safety factor of 10 (for intraspecies variability) seems unnecessary, because the minimal lethal dose already considers the most sensitive persons in a population.

The ARfD of 0.075 mg/kg body weight is valid for single oral ingestion of cyanides or HCN as well as for unprocessed foods with cyanogenic glycosides also containing the accompanying intact β-glucosidase. In case of these foods, the ARfD is relevant for a meal eaten within a short period of time, and a second meal with the same equivalent dose of cyanide may be possible on the same day after an interval of at least several hours, not leading to accumulation of cyanide in the body. For some foods, higher ARfD values may be possible, as demonstrated in this study for linseed. If such data are not available, a high content of cyanogenic glycosides in foods has to be treated like an equivalent content of free cyanide. In case of destroyed or missing specific β-glucosidase (persipan paste, isolated cyanogenic glycosides), the hazard potential is much lower.

In case of bitter apricot kernels, the highest possible levels of bound cyanide (4000 mg/kg in the literature: Zöllner and Giebelmann 2007; 3830 mg/kg was maximally measured in 12 halved kernels of our study) have to be considered for risk assessment, and the ARfD roughly corresponds to a maximum amount of 1.1 g kernels with 4.5 mg bound cyanide (three middle-sized or two big kernels). For fresh cassava roots, a maximum level bound cyanide of 10 mg/kg would be necessary in order to be also protective in case of large principal meal with 450 g. Such a value (10 mg/kg) is established for edible cassava flour (Codex Standard 176-1989, FAO/WHO 1995), but should also be protective for a single meal of unprocessed cassava constituting a higher hazard potential. In contrast, the often used cut-off of 50 mg/kg separating “sweet” and “bitter” cassava does not seem meaningful in terms of possible health risks, and even “sweet” cassava may require processing in case of consumption of a large meal. For persipan paste and linseed, considerations regarding risk assessment were already made above.

## Summary and conclusions

1. In terms of bioavailability of cyanide, its peak level in blood reflecting the rate of absorption is the most relevant parameter closely related to the (per)acute toxicity. In case of fast absorption of a bolus, the parameter is not significantly influenced by the rate of metabolism, which is relatively low and constant at relevant doses of cyanide (zero-order kinetics). Therefore, peak levels are defined by the volume of distribution primarily determined by the body weight. Any delay in absorption leads to lower peak levels and allows the body time for detoxification, which further contributes to lower peak levels.
2. The bioavailability of cyanide from foods with cyanogenic glycosides can maximally be equal to that of free cyanide. This requires a fast and complete destruction of the plant’s tissue structure by chewing (or other methods directly used before ingestion) and a high effectiveness of the plant’s specific β-glucosidase. Bitter apricot kernels and fresh cassava roots fulfil the last condition (the bound cyanide is as bioavailable as free cyanide), and life-threatening conditions and death are possible in case of consumption of the foods containing high doses of cyanide equivalents (Mlingi et al. 1992; Lasch and El Shawa 1981).
3. In case of inactivated or missing specific β-glucosidase (persipan paste, isolated cyanogenic glycosides), an ingestion of the same dose leads to much lower peak levels occurring relatively late after ingestion, as the enzymatic cleavage of cyanide can be achieved by bacterial flora of the gastrointestinal tract only leading to comparatively low rates of absorption. This applies to amygdalin, but has not been proved in humans for other types of cyanogenic glycosides. In case of linamarin, no significant bacterial cleavage was observed in a single person at all.
4. The hazard potential of other foods containing cyanogenic glycosides can be expected to lie in between the extremes described. This was demonstrated for linseed. Besides the content of bound cyanide, the effectiveness of the plant’s β-glucosidase as a specific property determines the hazard potential as a second important factor which may vary in individual cultivars (Chadha et al. 1995) or may change during post-harvest storage (Owiti et al. 2011). In the past, this factor was not considered in risk assessment of foods with cyanogenic glycosides.
5. Peak levels overproportionally increase with increasing doses, reflecting the decreasing proportion of detoxification per unit of time due to the constant metabolism rate (at higher doses). These nonlinear conditions contribute to the potential risk of consumption of foods with high content of cyanogenic glycosides, as a relatively little further increase in the dose may result in sudden occurrence of clinical symptoms. An evaluation of the area under the blood concentration–time curve (AUC) is not meaningful and no measure for the relative amount absorbed.
6. An ARfD of 0.075 mg/kg body weight was derived being valid for a single application/meal of alkalicyanides or HCN as well as of unprocessed foods with cyanogenic glycosides also containing the accompanying intact β-glucosidase. For some of these foods, this approach may be overly conservative due to slow release of cyanide. However, experimental data like that generated by this human study are required to assess the real hazard potential of such foods and to allow a meaningful risk assessment not requiring the use of high safety factors.

## Electronic supplementary material

Below is the link to the electronic supplementary material.

Supplementary material 1 (DOC 55 kb)

## Abbreviations

ARfD: Acute reference dose
*C*_max_: Maximum concentration in blood
*t*_max_: Time of occurrence of maximum concentration in blood
TST: Thiosulfate sulfurtransferase (rhodanese)
