# Supplementary material for: Bioavailability of cyanide after consumption of a single meal of foods containing high levels of cyanogenic glycosides: a crossover study in humans
Source: Arch Toxicol. 2015 Feb 24;90(3):559–74. doi: 10.1007/s00204-015-1479-8 (PMC4754328; doi:10.1007/s00204-015-1479-8)
Supplement: Supplementary file 1 — Supplementary material 1 (DOC 55 kb) [file 204_2015_1479_MOESM1_ESM.doc]

Abraham et al.

Bioavailability of cyanide after consumption of a single meal of foods containing high levels of cyanogenic glycosides: a crossover study in humans

**Supplementary Material**

**Alterations in the TST gene sequence of the twelve volunteers**

Genomic DNA was isolated from blood samples of the twelve volunteers of the study, and the coding region of the TST gene as well as 1 kb of the upstream promoter region was sequenced and analysed for alterations in comparison to the sequence of the TST gene region deposited in the NCBI database[[1]](#footnote-2). Sequence analysis revealed that three different single nucleotide polymorphisms (SNP) were present in the TST gene region of some of the volunteers. In all cases, the SNP was only present in one of the two alleles, meaning that the respective volunteer still contained one wild type allele and was therefore heterozygous for the respective SNP. The TST gene of one volunteer comprises the c.768C>T exchange that has already been described (Billaut-Laden et al. 2006) as a silent mutation in the TST coding region. Moreover, the TST sequence of three volunteers harbors a c.894*4G>A exchange four bases downstream of the TST stop codon. This mutation has also been described to have no effect on TST gene expression or rhodanese activity (Billaut-Laden et al. 2006). Finally, in the case of three volunteers, a heterozygous C>T exchange has been identified at position -210 of the TST gene with +1 being the first base of the TST start codon. Since the cyanide blood levels of these three volunteers were in the same range as those of the other volunteers, it is assumed that this (heterozygous) SNP in the TST promoter region did not significantly affect TST gene expression and in turn rhodanese-mediated cyanide detoxification. Taken together, TST genotyping did not give any indication for a mutation in the TST gene region of any of the volunteers that had to be considered in the evaluation of the study results.

**Supplementary Table 1**

Oligonucleotides and PCR conditions used for the amplification of the TST gene region of genomic DNA derived from blood samples of the volunteers of the study.

| Forward Primer | Reverse Primer | Annealing  Temperature | Region of the TST gene ^1)^ | PCR System |
| --- | --- | --- | --- | --- |
| TST-F1-2 | TST-1aR | 60°C | -1182 // -761 | FailSafe System ^2)^ |
| LF1F | TST-R1-2 | 60°C | -956 // -582 | FailSafe System |
| TST-F2-2 | TST-R2 | 60°C | -782 // -138 | GoTaq-Green ^3)^ |
| TST-F3 | TST-R3 | 60°C | -319 // +266 | GoTaq-Green |
| TST-F4 | TST-R4 | 60°C | +112 // +747 | GoTaq-Green |
| TST-F5 | TST-R5 | 60°C | +628 // +1221 | GoTaq-Green |
| LF2F | LF2R | 60°C | +7211 // +8201 | GoTaq-Green |

^1)^ The numbering refers to the first base of the TST start codon at position +1. Therefore, exon 1 of the TST gene covers bases +1 to +595, and exon 2 covers bases +7407 to +7705.

^2)^ FailSafe PCR System (Epicentre, Madison, WI)

^3)^ GoTaq Hot Start Green Master Mix (Promega, Madison, WI)

**Supplementary Table 2**

Sequences of the oligonucleotides used in this study (also see Supplementary Table 1)

| Name of the oligonucleotide | Sequence (5’ - 3’) |
| --- | --- |
| TST-F1-2 | CCCCCTCCAGAAAGGCAAG |
| TST-1aR | ATCGCGCCACCGCCAGAG |
| LF1F | GGCCGTGGGGGTACGCC |
| TST-R1-2 | ACTCTCGCAACGCTATCCTTC |
| TST-F2-2 | GGGGTTTGAACCGGCCAAC |
| TST-R2 | TTTACAGCAAGCCAGCGAGG |
| TST-F3 | TAACTTCTAGGAGCCAGATCCC |
| TST-R3 | TGTGGTTGCTGATGCCCAG |
| TST-F4 | CACCAGGCACCCGAGAGG |
| TST-R4 | TCCACTTTCTGGTTTTATCGATGG |
| TST-F5 | GTATTGCCCCATGGAAGGATAG |
| TST-R5 | AAACAGGGGCATTGAGCTTTG |
| LF2F | GGAGTTGCAGCATCTTAAGT |
| LF2R | CTCTATGGACATATTGGGAAAG |

1. (http://www.ncbi.nlm.nih.gov/nuccore/NC_000022.11?report=fasta&from=37009459&to=37021581&strand=true, accessed 09 April 2014) [↑](#footnote-ref-2)
